# Supplementary material for: Neural encoding with unsupervised spiking convolutional neural network
Source: Commun Biol. 2023 Aug 28;6:880. doi: 10.1038/s42003-023-05257-4 (PMC10462614; doi:10.1038/s42003-023-05257-4)
Supplement: Supplementary file 2 — Supplementary Information [file 42003_2023_5257_MOESM2_ESM.pdf]

Supplementary materials to "Neural encoding with unsupervised spiking convolutional neural network"

Chong Wang[1,2,3], Hongmei Yan[2,3,*], Wei Huang[2,3], Wei Sheng[2,3], Yuting Wang[2,3], Yun-Shuang Fan[2.3], Tao Liu[2], Ting Zou[2], Rong Li[2,3,*], Huafu Chen[1,2,3,*]

[1]The Center of Psychosomatic Medicine, Sichuan Provincial Center for Mental Health, Sichuan Provincial People's Hospital, University of Electronic Science and Technology of China, Chengdu, 611731, China

[2]School of Life Science and Technology, University of Electronic Science and Technology of China, Chengdu, 610054, China

[3]MOE Key Lab for Neuroinformation; High-Field Magnetic Resonance Brain Imaging Key Laboratory of Sichuan Province, University of Electronic Science and Technology of China, Chengdu, 610054, China

Email: chenhf@uestc.edu.cn, rongli1120@gmail.com, hmyan@uestc.edu.cn

Supplementary Table 1

The architecture of CNN used for neural encoding.

Operation	Output dimension
input	1×H×W
DoG layer	N×H×W
batch norm + maxpool 2×2	N×H/2×W/2
64 conv 5×5, str 1, pad 2	64×H/2×W/2
maxpool 2×2	64×H/4×W/4
fully connected layer	N_c

H: The height of the input image; W: The width of the input image; N: The number of DoG filters; N_c: The number of output classes.

Supplementary Table 2

The architecture of brain-optimized CNN (GNet) on natural image dataset.

Operation	Output dimension
input	1×128×128
DoG layer	6×128×128
batch norm + maxpool 2×2	6×64×64
64 conv 5×5, str 1, pad 2	64×64×64
batch norm +maxpool 2×2	64×32×32
8 conv 5×5, str 1, pad 2	8×32×32
maxpool 2×2	8×16×16
fully connected layer	number of voxels
