# Supplementary material for: Neural encoding with unsupervised spiking convolutional neural network
Source: Commun Biol. 2023 Aug 28;6:880. doi: 10.1038/s42003-023-05257-4 (PMC10462614; doi:10.1038/s42003-023-05257-4)
Supplement: Supplementary file 3 — Description of Additional Supplementary Files [file 42003_2023_5257_MOESM3_ESM.pdf]

Description of Additional Supplementary Files

File name: Supplementary Data 1
Description: The source data behind Fig. 2 in the paper.

File name: Supplementary Data 2
Description: The source data behind Fig. 4 in the paper.

File name: Supplementary Data 3
Description: The source data behind Fig. 5 in the paper.
