# Supplementary material for: Neural encoding with unsupervised spiking convolutional neural network
Source: Commun Biol. 2023 Aug 28;6:880. doi: 10.1038/s42003-023-05257-4 (PMC10462614; doi:10.1038/s42003-023-05257-4)
Supplement: Supplementary file 7 — Reporting Summary [file 42003_2023_5257_MOESM7_ESM.pdf]

nature portfolio

Corresponding author(s): COMMSBIO-23-0421

Last updated by author(s): Aug 6, 2023

Reporting Summary

Nature Portfolio wishes to improve the reproducibility of the work that we publish. This form provides structure for consistency and transparency in reporting. For further information on Nature Portfolio policies, see our Editorial Policies and the Editorial Policy Checklist.

Statistics

For all statistical analyses, confirm that the following items are present in the figure legend, table legend, main text, or Methods section.

n/a	Confirmed	
☐	☒	The exact sample size (n) for each experimental group/condition, given as a discrete number and unit of measurement
☐	☒	A statement on whether measurements were taken from distinct samples or whether the same sample was measured repeatedly
☐	☒	The statistical test(s) used AND whether they are one- or two-sided<br>*Only common tests should be described solely by name; describe more complex techniques in the Methods section.*
☒	☐	A description of all covariates tested
☒	☐	A description of any assumptions or corrections, such as tests of normality and adjustment for multiple comparisons
☐	☒	A full description of the statistical parameters including central tendency (e.g. means) or other basic estimates (e.g. regression coefficient) AND variation (e.g. standard deviation) or associated estimates of uncertainty (e.g. confidence intervals)
☒	☐	For null hypothesis testing, the test statistic (e.g. F, t, r) with confidence intervals, effect sizes, degrees of freedom and P value noted<br>*Give P values as exact values whenever suitable.*
☒	☐	For Bayesian analysis, information on the choice of priors and Markov chain Monte Carlo settings
☒	☐	For hierarchical and complex designs, identification of the appropriate level for tests and full reporting of outcomes
☐	☒	Estimates of effect sizes (e.g. Cohen's d, Pearson's r), indicating how they were calculated

Our web collection on statistics for biologists contains articles on many of the points above.

Software and code

Policy information about availability of computer code

Data collection	All the datasets used in this manuscript were publicly available online. The handwritten character dataset is publicly available at http://sciencesanne.com/research/, the handwritten digit dataset is publicly available at http://hdl.handle.net/11633/di.dcc.DSC_2018.00112_485, the grayscale natural image dataset is publicly available at https://crcns.org/datasets/vc/vim-1, the colorful natural image dataset is publicly available at https://github.com/KamitaniLab/GenericObjectDecoding.
Data analysis	The original datasets have been preprocessed. We built SCNN models using the SpykeTorch platform (https://github.com/miladmozafari/SpykeTorch), and other benchmark deep learning models were implemented using the Pytorch platform (https://pytorch.org/), the linear regression models were implemented using the scikit-learn platform (https://scikit-learn.org/).

For manuscripts utilizing custom algorithms or software that are central to the research but not yet described in published literature, software must be made available to editors and reviewers. We strongly encourage code deposition in a community repository (e.g. GitHub). See the Nature Portfolio guidelines for submitting code & software for further information.

Data

Policy information about availability of data

All manuscripts must include a data availability statement. This statement should provide the following information, where applicable:

- Accession codes, unique identifiers, or web links for publicly available datasets
- A description of any restrictions on data availability
- For clinical datasets or third party data, please ensure that the statement adheres to our policy

The handwritten character dataset is publicly available at http://sciencesanne.com/research/, the handwritten digit dataset is publicly available at http://hdl.handle.net/11633/di.dcc.DSC_2018.00112_485, the grayscale natural image dataset is publicly available at https://crcns.org/datasets/vc/vim-1, the colorful natural image dataset is publicly available at https://github.com/KamitaniLab/GenericObjectDecoding.

Human research participants

Policy information about studies involving human research participants and Sex and Gender in Research.

Reporting on sex and gender	Sex effects was not considered in this study.
Population characteristics	The four image-fMRI datasets adopted in this study contain 11 participants in total, and all of them were healthy and had normal or corrected-to-normal vision.
Recruitment	No participant was recruited in this study
Ethics oversight	All participants in the handwritten character dataset gave written consent according to the institutional guidelines set forth by the local ethics committee (CMOregion Arnhem–Nijmegen, The Netherlands) before the experiment. The handwritten digit dataset was approved by the ethics consortium from Donders Institute for Brain, Cognition and Behaviou. The experiment used in the grayscale natural image dataset were approved by the University of Caifornia, Berkeley Committee for the Protection of Human Subjects. All subjects in the colorful natural image dataset provided written informed consent for participation in the experiments, and the study protocol was approved by the Ethics Committee of ATR.

Note that full information on the approval of the study protocol must also be provided in the manuscript.

Field-specific reporting

Please select the one below that is the best fit for your research. If you are not sure, read the appropriate sections before making your selection.

☒ Life sciences ☐ Behavioural & social sciences ☐ Ecological, evolutionary & environmental sciences

For a reference copy of the document with all sections, see nature.com/documents/nr-reporting-summary-flat.pdf

Life sciences study design

All studies must disclose on these points even when the disclosure is negative.

Sample size	The handwritten character dataset comprises the fMRI data from 3 participants while viewing 360 stimuli; the handwritten digit dataset comprises the fMRI data from 1 participants while viewing 100 stimuli; the grayscale natural image dataset comprises the fMRI data from 2 participants while viewing 1870 stimuli; the colorful natural image dataset comprises the fMRI data from 5 participants while viewing 1250 stimuli.
Data exclusions	No data was excluded from the analysis.
Replication	Our approach was verified on four different datasets, and we repeated our approach twice on the grayscale natural image dataset to test the reproducibility.
Randomization	No randomization is performed.
Blinding	No blinding is done.

Reporting for specific materials, systems and methods

We require information from authors about some types of materials, experimental systems and methods used in many studies. Here, indicate whether each material, system or method listed is relevant to your study. If you are not sure if a list item applies to your research, read the appropriate section before selecting a response.

Materials & experimental systems

n/a	Involved in the study
☒	☐ Antibodies
☒	☐ Eukaryotic cell lines
☒	☐ Palaeontology and archaeology
☒	☐ Animals and other organisms
☒	☐ Clinical data
☒	☐ Dual use research of concern

Methods

n/a	Involved in the study
☒	☐ ChIP-seq
☒	☐ Flow cytometry
☐	☒ MRI-based neuroimaging

Magnetic resonance imaging

Experimental design

Design type: task-state fMRI; The handwritten character and grayscale natural image datasets were event designed, and the handwritten digit and colorful natural image datasets were block designed.

Design specifications: The experiment designs of the datasets were described in their original publications (Schoenmakers et al., 2013; Van Gerven et al., 2010; Kay et al., 2008; Horikawa et al., 2017).

Behavioral performance measures: Subjects in the colorful natural image dataset performed a one-back repetition detection task on the images, responding with a button press for each repetition to maintain their attention on the presented images (mean task performance across five subjects sensitivity=0.930; specificity=0.995). No behavioral tasks were performed in other datasets.

Acquisition

Imaging type(s): functional

Field strength: 3 Tesla

Sequence & imaging parameters: The sequence and imaging parameters of the datasets were provided in their original publications (Schoenmakers et al., 2013; Van Gerven et al., 2010; Kay et al., 2008; Horikawa et al., 2017).

Area of acquisition: The visual cortex, visual areas were localized using the standard retinotopy experiment.

Diffusion MRI: ☐ Used ☒ Not used

Preprocessing

Preprocessing software: SPM5/SPM8

Normalization: The data were not normalized because our analysis was performed on the individual level.

Normalization template: The data were not normalized.

Noise and artifact removal: The fMRI data underwent head motion correction using SPM.

Volume censoring: No volume censoring was performed.

Statistical modeling & inference

Model type and settings: Two sample t-test

Effect(s) tested: We performed the two sample t-test to compare the prediction accuracies of different encoding models.

Specify type of analysis: ☐ Whole brain ☒ ROI-based ☐ Both

Anatomical location(s): V1, V2, and V3

Statistic type for inference (See Eklund et al. 2016): voxel-wise

Correction: We did not made multiple comparisons in this study.

Models & analysis

n/a	Involved in the study	
☒	☐	Functional and/or effective connectivity
☒	☐	Graph analysis
☐	☒	Multivariate modeling or predictive analysis

Multivariate modeling and predictive analysis

We used the spiking convolutional neural network to extract the visual features of the input image, and built a linear regression model for each voxel to predict its fMRI response.
